# Nanomechanical properties of α-synuclein amyloid fibrils: a comparative study by nanoindentation, harmonic force microscopy, and Peakforce QNM

**DOI:** 10.1186/1556-276X-6-270

**Published:** 2011-03-30

**Authors:** Kim Sweers, Kees van der Werf, Martin Bennink, Vinod Subramaniam

**Affiliations:** 1 Nanobiophysics Group, MESA+ Institute for Nanotechnology, Faculty of Science and Technology, University of Twente, Enschede, The Netherlands

## Abstract

We report on the use of three different atomic force spectroscopy modalities to determine the nanomechanical properties of amyloid fibrils of the human α-synuclein protein. α-Synuclein forms fibrillar nanostructures of approximately 10 nm diameter and lengths ranging from 100 nm to several microns, which have been associated with Parkinson's disease. Atomic force microscopy (AFM) has been used to image the morphology of these protein fibrils deposited on a flat surface. For nanomechanical measurements, we used single-point nanoindentation, in which the AFM tip as the indenter is moved vertically to the fibril surface and back while the force is being recorded. We also used two recently developed AFM surface property mapping techniques: Harmonic force microscopy (HarmoniX) and Peakforce QNM. These modalities allow extraction of mechanical parameters of the surface with a lateral resolution and speed comparable to tapping-mode AFM imaging. Based on this phenomenological study, the elastic moduli of the α-synuclein fibrils determined using these three different modalities are within the range 1.3-2.1 GPa. We discuss the relative merits of these three methods for the determination of the elastic properties of protein fibrils, particularly considering the differences and difficulties of each method.

## Introduction

Amyloid fibrils are insoluble protein aggregates that have been associated with a range of neurodegenerative diseases, including Huntington, Alzheimer's, Parkinson's, and Creutzfeldt-Jakob disease [1]. The fibrils typically have a diameter ranging from 4 to 12 nm, and lengths from 100 nm up to several microns [2-4]. In this study, we investigated the nanomechanical properties of amyloid fibrils formed from the human α-synuclein protein, which is associated with Parkinson's disease. α-Synuclein amyloid fibrils are found in the brains of Parkinson's disease patients as components of larger plaques called Lewy bodies [5,6].

Atomic force microscopy (AFM) has been primarily used as an imaging tool to determine morphological parameters such as height and length of amyloid fibrils, such as those formed from α-synuclein [2-4], insulin [7], and β-lactoglobulin [8]. AFM is also a powerful technique for characterizing mechanical properties. With the ability to exert and measure forces up to the piconewton range, AFM is a particularly suitable tool to determine the nanomechanical properties of nanometer-sized biological structures, such as amyloid fibrils. Mechanical properties such as stiffness, rigidity, resistance to breakage or adhesive properties of these fibrils or individual monomers are interesting for the use of these fibrils as nanomaterials, for getting a better understanding of the physico-chemical properties of these fibrils, and to get more insight into their structure and growth [9-14].

Indentation-type AFM or single-point nanoindentation (SPI), for example, implemented as 'Point-and-Shoot' in the Veeco operating software, is the most widely used method to measure nanomechanical properties of a sample. In this mode, the tip approaches and indents the sample until a certain predefined force is reached. At this point the tip is retracted again. During this approach and retract cycle the force is continuously measured, resulting in a force versus distance graph. AFM nanoindentation has been performed on different biological substrates such as collagen [15], insulin fibrils, and crystals [16], but also on different polymeric materials, such as fibrils used for biodegradable scaffolds [17]. The approach-retract cycle is typically performed at a rate of 0.5 to 10 Hz, which makes this method inherently slow. To get an overview of the mechanical properties of a sample, nanoindentation can be used in a force-volume mode. Here, for every pixel in an image a complete force curve is recorded, which results in data acquisition times of up to hours for a single image.

Recently, several different surface property mapping techniques have become available that work at much higher speeds, leading to significantly increased data throughput [18-20]. Two commercially available approaches are PeakForce QNM and Harmonic force microscopy or HarmoniX (Veeco, Santa Barbara, CA, USA). PeakForce QNM is based on the force-volume approach; however, the speed of taking the force curves is significantly increased (either at 1 or 2 kHz). In this mode the maximum force exerted on the sample is maintained constant, which is beneficial for soft delicate biological samples. Because of the recent introduction of the Peakforce QNM method, only a few studies have been reported, such as the stiffness mapping of polymer blends [18].

HarmoniX is another surface property mapping technique based on the nonlinear dynamic behavior of a cantilever in tapping mode due to repulsive and attractive forces caused by the specific material characteristics of the sample acting on the tip [21,22]. Because of the low bandwidth of the cantilever response, this information ends up in the phase image as obtained during tapping mode imaging. This phase signal is related to energy dissipation, which is determined by the viscoelastic and adhesive properties of the sample [21,23]. However, because of the convolution of multiple physical properties into one signal, interpretation of these images is not straightforward. The higher harmonic vibrations of the cantilever excited by these material properties can provide more information, but they are heavily suppressed and are difficult to measure [21,24]. In HarmoniX, a torsional cantilever with the tip positioned off-axis solves this problem and acts as a high bandwidth force sensor [24]. HarmoniX has been applied to both polymers and biological features, for example, DNA [25,26].

We used these three different methods, SPI, PeakForce QNM, and HarmoniX, to determine the modulus of elasticity of protein nanofibrils, generated from the E46K mutant of the human α-synuclein protein. The resulting values for the elastic modulus are in the range between 1.3 and 2.1 GPa. We discuss the relative merits of the application of these three methods specifically for the determination of the elastic properties of protein fibrils in more detail, with particular emphasis on the differences and difficulties of each method.

## Results

### Single-point nanoindentation experiments in liquid

**Figure 1:**
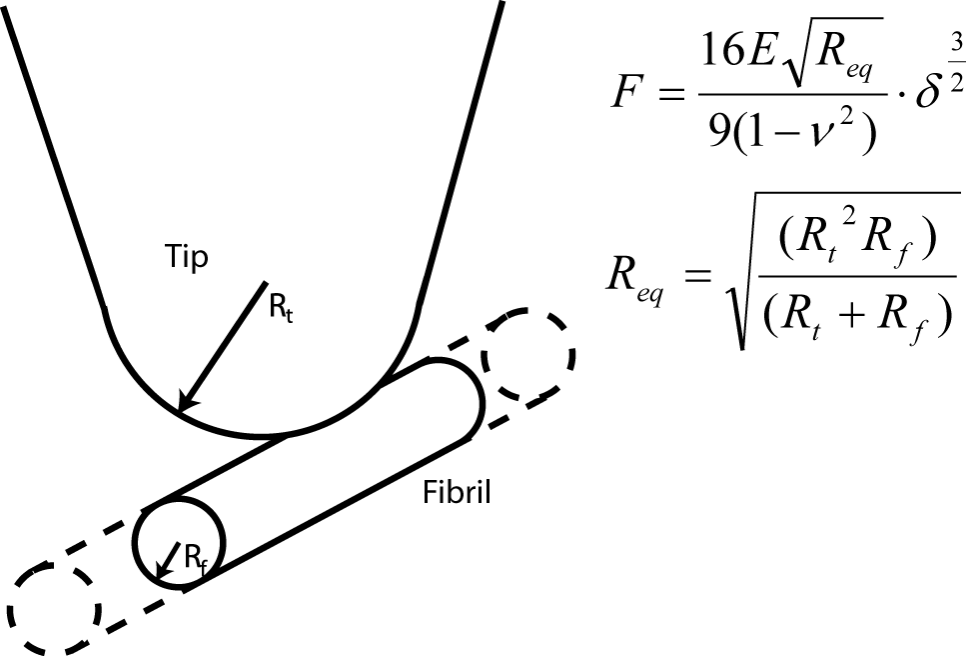
**Schematic representation of equivalent contact radius**. Schematic representation of the AFM tip as a spherical indenter and the protein fibril as an infinitely long cylinder.

α-Synuclein fibrils deposited on mica were scanned both in tapping mode and contact mode, respectively, for determining the height and finding the indentation points for the SPI measurements. We determined an average fibril height of 9.0 ± 0.4 nm (*N *= 60) from the tapping mode images. This average height value was used to determine the effective contact surface in the indentation measurements according to the model shown in Figure 1. The fibril heights measured in contact mode imaging were considerably lower and were therefore not used in determining the average fibril height. This was attributed to the pressure from the tip on the sample. The force exerted on fibrils with the 0.1 N/m cantilever during scanning was between 0.5 and 1 nN.

**Figure 2:**
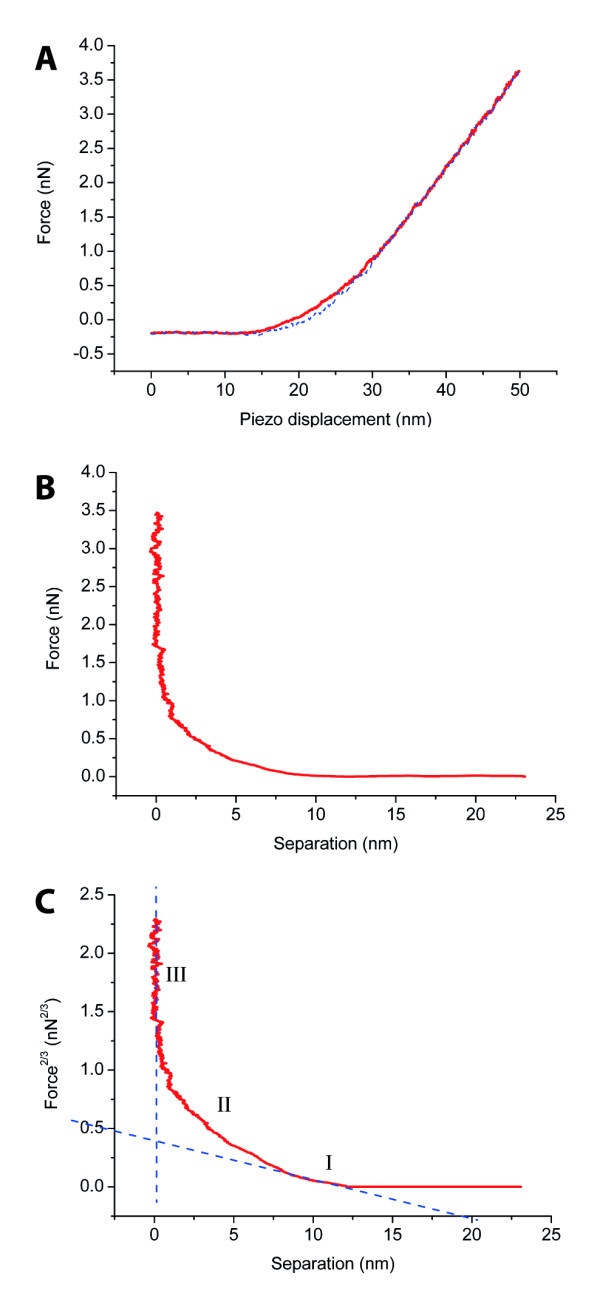
**Typical force curves**. **(A) **A typical force versus piezo displacement curve obtained from the measurement, with the approach curve (solid red) and the retract curve (dashed blue). **(B) **Force versus separation approach curve calculated from the force versus piezo displacement curve. **(C) **Force to the power of 2/3 versus separation approach curve, showing distinct transition from the tip only sensing the fibril (part I) to the part where the tip is sensing the mica under the fibril (part II) until the part where the tip is only pressing on the mica (part III). From the slope of part I, a modulus of elasticity of 1.2 GPa was calculated for the force curve presented here.

We performed nanoindentation experiments on five fibrils, each of which was indented 8 times at different locations along its length. A typical force distance curve resulting from this procedure is shown in Figure 2A. The absence of adhesion during the measurements allowed the use of the Hertz model.

Although every fibril was indented 8 times, not all curves were suitable for analysis. For some curves, the *r*^2 ^values of the linear fit did not exceed 0.95 in the part where the tip was indenting the fibril (part 1 in Figure 2C). From the curves that were analyzed, an average elastic modulus of 1.3 ± 0.4 GPa (*N *= 31) was found for α-synuclein fibrils.

### Harmonic force microscopy

**Figure 3:**
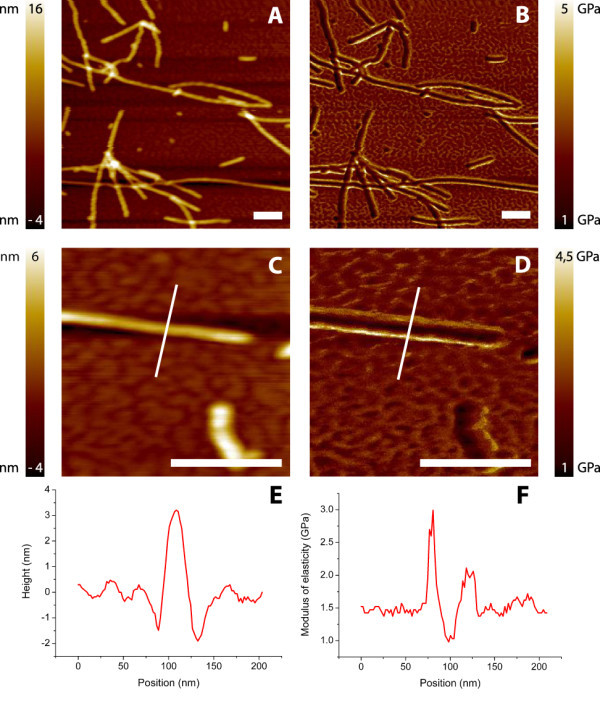
**Harmonic force microscopy images**. Height **(A, C) **and corresponding elasticity images **(B, D) **of α-synuclein fibrils on mica. **E **represents the cross-sections drawn over the fibril in **C**. **F **represents the cross-section from **D **and shows a few scan artifacts. The background, mica, has here a stiffness of ± 1.5 GPa, probably caused by the limited range of elastic moduli which can be measured with the chosen cantilever. The peaks shown around 80 and 120 nm are edge effects caused by changing contact areas. The dip around 100 nm is assumed to be relevant for averaging and used to determine a modulus of elasticity. Scale bars are 250 nm.

A sample of α-synuclein fibrils deposited on mica was scanned. Figure 3 shows two typical images recorded, with corresponding height and elasticity profiles. The fibrils show considerably lower modulus of elasticity compared to the background. However, the edges of the fibril show increased modulus of elasticity values, also displayed in the cross-section of the fibril shown in Figure 3F. We attribute this effect is due to the changing contact area compared to the contact area shown in Figure 1 where the tip is indenting the middle of the fibril. This artifact is also visible in the height images derived from the harmonic force mode, shown in Figure 3E, and they are therefore not used in further analysis. For each individual fibril, the values for the elastic modulus measured along the fibril were averaged. The average value was 1.2 ± 0.2 GPa (*N *= 95).

### Peakforce QNM

**Figure 4:**
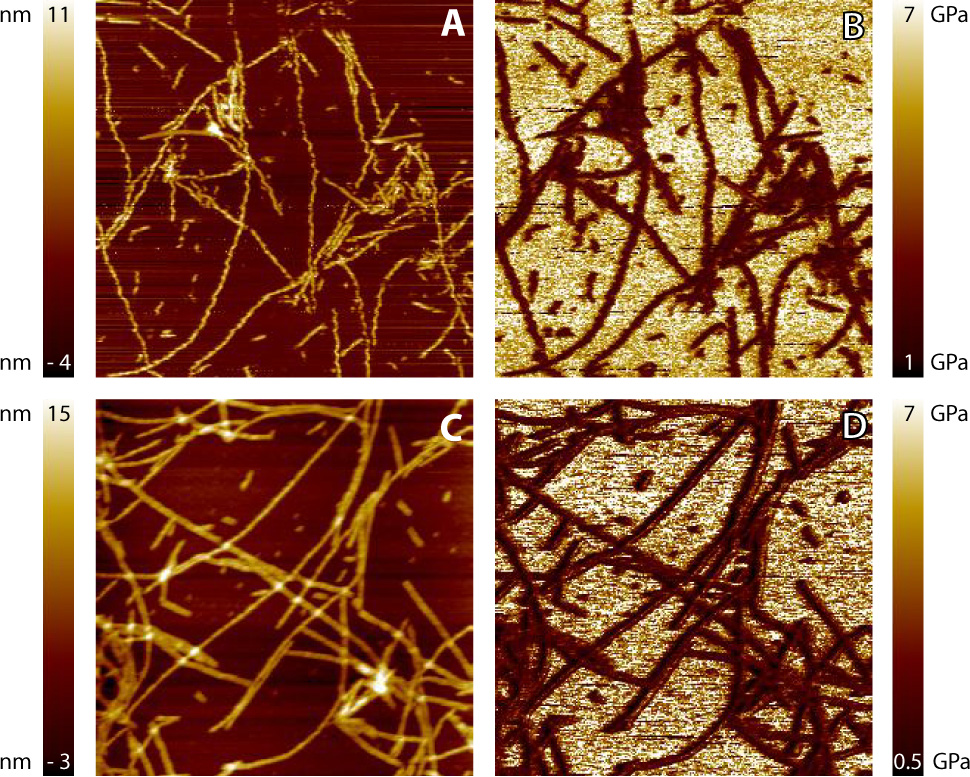
**Peakforce QNM images in liquid and ambient conditions**. Height **(A, C) **and corresponding elasticity maps **(B, D) **recorded with Peakforce QNM. Panels **A **and **B **are recorded in liquid (setpoint is 14 nN) and **C **and **D **in ambient conditions (setpoint is 16 nN). The fibrils have in these images an average modulus of elasticity of 3 GPa and mica between 6 and 7 GPa. Image size is 2 × 2 μm.

The surface property mapping technique Peakforce QNM is able to image the sample both in ambient conditions and in buffer solution. Figure 4 shows height images and the corresponding elasticity maps obtained with Peakforce QNM of α-synuclein fibrils, obtained in buffer (Figure 4A, B) and in air (Figure 4C, D). These images were obtained with a high setpoint of around 15 nN and show that for both liquid and ambient conditions the height and elasticity ranges which can be obtained with Peakforce QNM are similar. However, this large setpoint causes the fibrils to break, especially in liquid, see Figure 4A.

**Figure 5:**
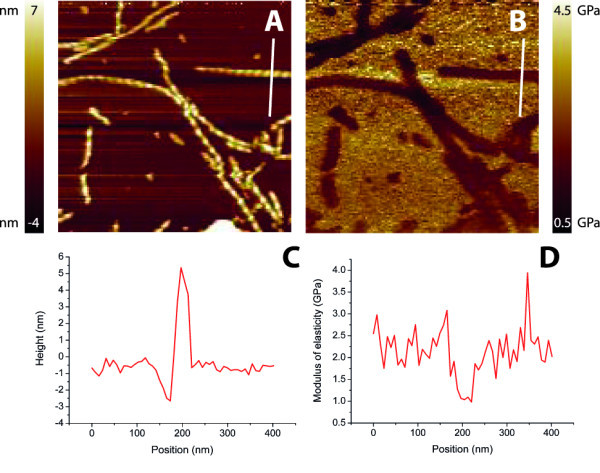
**Peakforce QNM images**. Height and stiffness map of fibrils obtained with a setpoint of 1 nN in liquid, images size is 1 μm **(A, B)**. **C **and **D **represent the cross-section of the fibril. Notice that in Peakforce QNM the artifacts at the edges of the fibrils seen in HarmoniX (Figure 3F) caused by changing contact areas are absent.

To prevent damage to the α-synuclein fibrils, a lower setpoint of 1-2 nN was used. This resulted in intact fibrils with significant lower values of the elastic moduli (Figure 5). The elastic modulus for each fibril is determined from the average value of the DMT modulus obtained along the fibril length. This resulted in a modulus of elasticity of 1.3 ± 0.3 GPa (*N *= 57) for the fibrils in ambient conditions and 1.0 ± 0.2 GPa (*N *= 59) for those in liquid.

## Discussion

### Choosing the right cantilever

**Figure 6:**
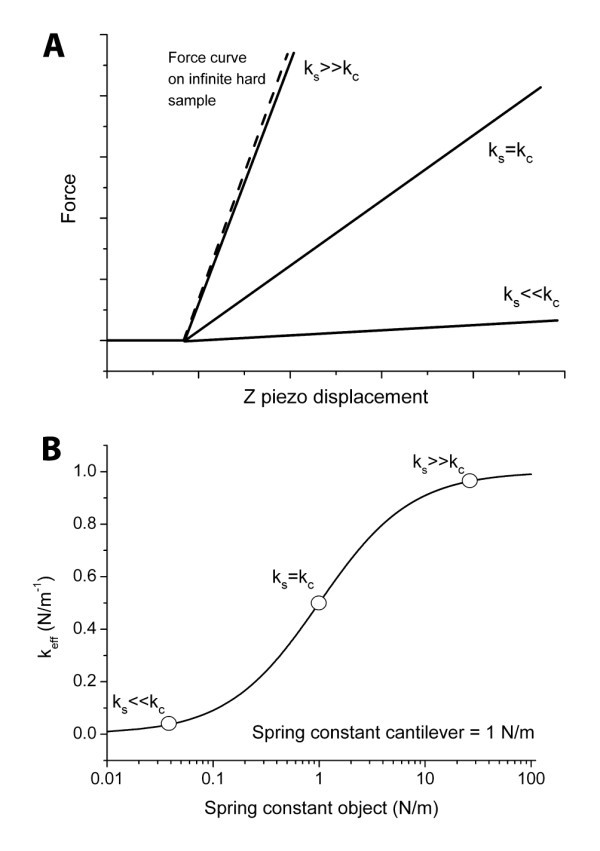
**Effective spring constant as a function of sample stiffness**. **(A) **Force versus *z *piezo displacement curve in case of sample spring constant larger, the same or lower compared to the spring constant of the cantilever. **(B) **Effective spring constant (*k*_eff_, representing the slope of the force curves in **A) **as a function of the stiffness of the sample. From the slope of this curve it is clear that the maximum sensitivity is achieved when both spring constants are of the same order of magnitude.

In order to measure the elastic properties of a material, the choice of the cantilever is key. In nanoindentation the highest sensitivity (and thus accuracy) is achieved if the spring constant of the probe cantilever is identical to the effective spring constant of the sample (also referred to as contact stiffness), see Figure 6. If the spring constant of the cantilever is more than 10 times lower or higher than that of the sample, the sensitivity is about 3 times lower, see Figure 6 making the determination of the elastic modulus less accurate. Practically since one does not know the stiffness of the sample *a priori*, an estimation is necessary. This is also the case for the surface mapping methods. The nominal elastic modulus ranges accessible by HarmoniX and Peakforce QNM are 10 MPa-10 GPa and 0.7 MPa-70 GPa, respectively [18]. However, as noted above, this range depends on the cantilever that is used for the measurements and is in practice significantly smaller.

A second point to consider when choosing the cantilever is the adhesion between the tip and the sample. The spring constant of the cantilever should be sufficiently high to create enough force to come loose from the surface. In the PeakForce QNM experiments reported here on protein fibrils, performed in ambient air, an adhesion of few nanonewtons was observed. For reproducible and proper deflection curves in air we used in this case a cantilever with a spring constant of approximately 27 N/m. In the HarmoniX mode the fibrils are measured in a special tapping mode. In this mode reproducible results were obtained with cantilevers with medium stiffness of 2 N/m in ambient conditions. The cantilever used for the nanoindentation measurements (0.1 N/m) showed an incredibly large artifact in both approach and retract curves at the 1 kHz ramp rate in Peakforce QNM in liquid, which was not seen in the nanoindentation measurements. This artifact could be induced by the impact of the effective mass and damping forces at the working frequency of 1 kHz. These hydrodynamic forces acting on the cantilever are frequency-dependent [27,28]. Although we do not know how the Peakforce QNM software compensates for this, it is possible these effects in liquid could interfere with the measurements. Scanning with stiffer cantilevers with nominal spring constant 2.8 N/m yielded reproducible results.

Finally, in addition to choosing the optimal cantilever stiffness, it is also important to ensure that the resonance frequency for Peakforce QNM imaging is above 10 kHz, in order not to interfere with the 1 kHz ramping.

### Calibration

The calibration of all three methods is difficult and consists of several steps. For all methods one needs the deflection sensitivity, the spring constant of the cantilever and the tip radius. For the SPI experiments the tip radius can be determined afterwards. Both surface mapping methods need the tip radius as an input parameter before measuring. For HarmoniX, in addition to this tip radius, some additional parameters, such as the torsional frequency, are needed. An alternative way of calibration of the surface mapping methods was done with the reference sample (see "Methods" section). In this study, this reference sample is only used in the HarmoniX measurements.

### Analysis of results

#### Error analysis

All three techniques use a contact mechanics model which is based on assumptions and parameters which can only be determined with a limited accuracy. The first assumption starts with the Poisson ratio for these protein fibrils. For small biological samples this ratio between lateral strain and axial strain is not known. The theoretical upper limit is 0.5 and concrete as a material has a value between 0.1 and 0.2. In this study we used 0.3, because we assumed the fibrils to be in the same range as polymers [29]. This Poisson ratio has only a small influence on the actual modulus of elasticity values (Poisson ratio change from 0.3 to 0.4 gives a 5% change in modulus of elasticity).

**Figure 7:**
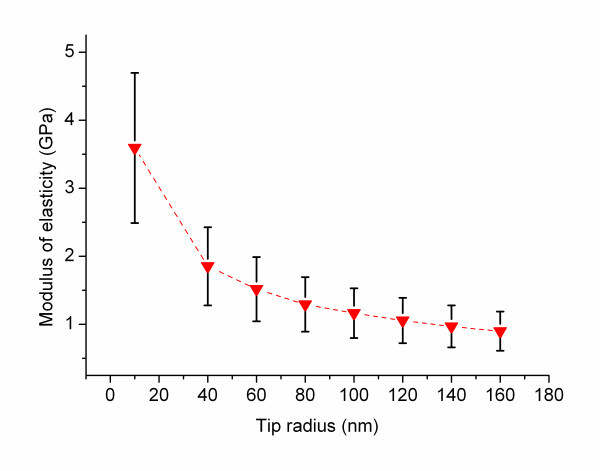
**Dependence of modulus of elasticity in tip radius**. The force curve data obtained with SPI measurements are used to calculate the modulus of elasticity with variable tip radii while all other parameters are kept constant.

The tip radius has, compared to the Poisson ratio and fibril radius, a large impact on the results. It is therefore important to measure the tip radius after the experiments. The tip radius is in our experience in practice always larger than the manufacturer specification, both before and after the experiment. Figure 7 shows the impact of the tip radius on the results of the SPI measurements on the α-synuclein fibrils when all the other parameters are kept constant. The dependence is less significant at larger tip radii.

However, even when using blunt tips there are measurement errors which have to be considered. The radius of the tip in indentation studies is often determined by scanning electron microscopy [16] after the indentation experiments where the tip shape could be influenced by wear [30]. Another method is to determine the tip radius from tip sample convolution models [21,31]. From previous studies the error from the tip sample convolution method is around 30% [32].

An additional important step is the calibration of the cantilever spring constant. There are a number of techniques available to determine the spring constant, each with their own uncertainties [33-35]. In this study, cantilevers were calibrated with the thermal noise method, which has an associated average error of 5% [33].

All three methods described here are susceptible to relatively large systematic errors arising from the compounding of errors inherent to the different calibration and characterization techniques. Using the law of propagation of errors, we estimate that this systematic error combined with the above-mentioned and the previously described 2% error in the deflection sensitivity measurements [34] yields an uncertainty of approximately 39% of the average measured value. In addition to these errors, experimental data are also influenced by the expected statistical variation due to heterogeneity of a large sample set.

#### Finite sample thickness

When indenting small fibrillar features with a relatively large tip radius one has to take the finite sample thickness effects into account. As shown in Figure 2C the force curve displays distinct regimes: from being free in air above the sample to the initial fibril indenting section where the tip 'only' feels the fibril (Figure 2C, part I), to where the mica underneath starts to play a role (Figure 2C, part II), and finally to the last section where only the hard surface is felt by the tip (Figure 2C, part III). The initial 20% of the total height of a feature is thought to be unaffected by these final sample thickness effects for large objects relative to the tip radius [12]. However, at the typical size scales of these nanofibrils, a correction for these effects is necessary [16,36]. In the Peakforce QNM software these effects are not considered and therefore not compensated for [18]. In HarmoniX there is also no correction for these effects. However, the question is whether these effects are nearly as pronounced in HarmoniX because of the small indentations that are made with this technique. In the SPI measurements from this study the correction factor was small (~1.3) because of the rather high modulus of elasticity.

#### Discussion of results

All methods used in this study yielded moduli of elasticity between 1.3 and 2.1 GPa (see Table 1), which is close to values found for collagen and other amyloid fibrils [10,15]. These values are somewhat smaller than those obtained for films made with fibrillar networks of β-lactoglobulin (5.2-6.2 GPa) and lysozyme (6.7-7.2 GPa) [37], and potentially reflect the differences in experimental conditions. We also measured insulin and lysozyme amyloid fibrils using HarmoniX under ambient conditions. The values measured, 1.4 ± 0.2 GPa for lysozyme fibrils and 1.4 ± 0.1 GPa for insulin, were commensurate to that measured for α-synuclein. The modulus previously found for insulin fibrils measured with SPI in liquid, which is at a lower working speed, is three orders of magnitude lower [16]. However, Smith et al. [11] have found a value of 3.3 GPa for insulin fibril using force spectroscopy on suspended fibrils. Note that in this work all the methods result in relatively similar values, although they all have very different operation speeds.

**Table 1:** Overview of results from different methods

Method	Environment	Operation frequency (Hz)	Uncorrected modulus of elasticity (GPa)	Modulus of elasticity (GPa)
Nanoindentation	Liquid	1	-	1.3 ± 0.4
Peakforce QNM	Liquid	10^3^	1.0 ± 0.2	1.6 ± 0.3
Peakforce QNM	Air	10^3^	1.3 ± 0.3	2.1 ± 0.5
HarmoniX	Air	10^5^	1.2 ± 0.2	1.9 ± 0.3

Overview of results from different methods under different environmental conditions, 4th column represents the corrected values for modulus of elasticity. This correction is the same as done in the analysis of the indentation measurements; the contact area is changed to a spherical indenter on an infinite cylinder (average fibril height of 9.0 ± 0.4 nm, measured in AFM tapping mode) and is corrected for the finite sample thickness as described in the "Methods" section.

The spread in the SPI measurements is also comparable to earlier work. The reason for this spread, besides the previously mentioned errors, has been related to heterogeneity in the internal packing of amyloids [1,16,38].

Both surface contact area and finite sample thickness corrections were performed offline on the HarmoniX and Peakforce QNM data, see Table 1 which results in higher values. The finite sample correction value found in the analysis of the SPI of 1.3 and the relation for a spherical indenter on an infinite long cylinder are used. The high modulus of elasticity of the fibrils suggests a high packing density. The difference between liquid and ambient air conditions becomes more significant after correction. With the uncorrected values the difference is lower, which suggests little room for water within the fibril, but the corrected results could point to an observed drying effect.

However, the large spread, seen in Table 1 and in previous studies, combined with the large systematic error of 39% calculated above makes interpreting these results very difficult.

## Conclusions

The nanometer scale diameters of α-synuclein protein fibrils pose some serious challenges for interpretation of the data obtained with SPI, HarmoniX and Peakforce QNM. The typical size scales of the fibrils give rise to finite sample thickness effects [16,36]. Furthermore, these fibrils cannot be described as a flat film on a surface for which all the standard models are valid [39,40]. Finally, these samples have strong adhesive properties which results in choosing cantilevers that possibly result in less contrast between the fibrils and the surface, because of the mismatch between cantilever and sample stiffness. All these difficulties are addressable with the conventional nanoindentation measurements, where the analysis is mostly done offline and in custom-written algorithms. For the surface property mapping techniques it is at this point only possible to customize the analysis in a limited manner. The methods come with specific conditions in which the analysis is valid. First, the tip should be a hard sphere compared to the sample. Second, only elastic deformation is taken into account. Last, the sample should not be confined vertically (finite sample thickness effect) or laterally (by surrounding material) [18]. For protein fibrils the second condition is not actually known, after indentation with high forces (> 3 nN) the fibrils appear to be broken, while with lower forces they stay intact (< 2 nN). The third condition is not met in case of the protein fibrils. For HarmoniX it is also good to keep in mind that theoretically one needs an infinite number of frequency components to reconstruct the real time interaction between the tip and the surface [18].

To obtain in a short amount of time quantitative modulus of elasticity for protein fibrils the surface property methods are relatively easy to use and fast. However, recording individual curves on the fibrils during scanning is necessary to analyze the curves for all the conditions that are not met in these methods. In case of the measurements done on the protein fibrils the differences are within each others error ranges. This may not be the case for other biological structures. It is essential to understand the limitations of each method and carefully analyze the data, including the individual force curves, according to the valid conditions for the specific structures.

## Methods

### Sample preparation

E46K disease mutant α-synuclein was recombinantly expressed and purified as previously described [4]. A 100 μM monomeric E46K solution in 10 mM Tris-HCl, 50 mM NaCl, pH 7.4 was incubated at 70°C in Eppendorf tubes under constant shaking. After 27 h, well-defined protein fibrils were formed in solution, which was verified by a Thioflavin T fluorescence assay specific for cross-beta structures characteristic of amyloid fibrils.

Samples for AFM imaging in liquid were prepared by placing 50 μl of a 5× diluted solution containing fibrils on the mica substrate. This solution was allowed to adsorb for 10 min and then washed gently with 200 μl buffer. For imaging, 80 μl of fresh buffer solution was placed on the sample. We used the same buffer solution (10 mM Tris-HCl, 50 mM NaCl, pH 7.4) for both dilution and imaging. For the measurements performed in ambient air, a 10× diluted protein solution was placed on mica substrates and allowed to adsorb in the same manner as described above. Subsequently, the sample was washed with 200 μl milliQ water and dried with a gentle nitrogen stream.

### AFM cantilever and tip characterization

The tip radius was determined with two different methods. First, from the AFM height images of protein fibrils the tip radius was derived from the fibril height-to-width ratio based on tip-sample convolution [21,31]. Only fibrils that were perpendicular to the scan axis were used. From the tip sample convolution method an average tip radius of 100 nm was determined. Second, the tip was imaged by scanning electron microscopy (Philips XL30 ESEM-FEG). With the SEM, the average tip radius was found to be approximately 80 nm. For both methods, the tip resulted in a considerably larger number than the nominal tip radius provided by the manufacturer. An average value of 90 nm was used in the analysis with an error of 30%.

The cantilever spring constants were determined with the thermal noise method implemented in the Veeco software and were assumed to have a 5% error [33].

### Single-point nanoindentation

A Bioscope II microscope (Veeco, Santa Barbara, CA, USA) was used for the SPI experiments. In order to measure the fibril heights, AFM tapping mode images were recorded in a physiological buffer (10 mM Tris-HCl, 50 mM NaCl, pH 7.4) in tapping mode with low force settings (reduced to 3 nm, 80-90% of the free amplitude) to minimize interaction with the sample. We use silicon nitride probes (MSCT, tip F, 0.5 N/m, Veeco, Santa Barbara, CA, USA) for these measurements. The average fibril height measured in tapping mode is used to determine the surface contact area for all three indentation methods. The indentation measurements were performed with the "Point and Shoot" application in the NanoScope 7.30 (Build R2Sr1.) software. To locate the indentation locations we first imaged the fibrils in contact mode using another probe (MSCT, tip E, 0.1 N/m, Veeco, Santa Barbara, CA, USA). This probe was selected to, on one hand, minimize the forces during contact mode imaging and, on the other hand, to match the spring constant of the cantilever to the stiffness of the sample for the indentation measurements. Every fibril was indented approximately 8 times at different positions along its length. Prior to fibril indentation, force curves were recorded on the mica substrate close to the fibril to determine deflection sensitivities of the cantilevers.

### Data analysis

The raw deflection curves, obtained in the SPI mode, were converted to a force separation curve using the deflection sensitivities and the spring constants of the cantilevers in a custom written Matlab program. To extract the elastic modulus from the force separation curve, the Hertz model was used to analyze the force curve [39]. This model, in the case of a spherical indenter on a cylinder shaped object, is given in Figure 1 where *F *is the load, *v *the Poisson ratio, δ the separation, and *E *the modulus of elasticity. The equivalent contact radius *R*_eq _for a spherical indenter with radius *R*_t_, with an infinitely long cylinder with radius *R*_f _is given by the expression in Figure 1. The modulus of elasticity was determined from the slope of the curve where *F*^2/3 ^was plotted versus the separation. Small segments along this curve were fitted to a linear equation and the *r*^2 ^value was determined for every fit, yielding an elastic modulus as a function of separation. From the point the force increases, the *r*^2 ^value increases and only fits with an *r*^2 ^above 0.95 were used in the analysis. From the point of contact the modulus of elasticity values for the following 2 nm were averaged (20% indentation [12]).

Due to the finite thickness effects, the obtained modulus of elasticity is influenced by the stiff underlying substrate (mica). A correction factor for this effect was applied which was a function of the maximum applied force and the value of the uncorrected modulus of elasticity [16,36].

All analysis steps were implemented in a custom Matlab program. The algorithm analyzes both the force curve and the *r*^2 ^curve to accurately determine the point-of-contact, that is, the separation at which the tip starts indenting the fibril. This point is defined as the point where the force distance curve leaves the baseline, and *r*^2 ^adopts a value higher than 0.95.

### Harmonic force microscopy

HarmoniX was performed under ambient conditions (that is, at room temperature without further control of humidity) on a Veeco Multimode microscope with a Nanoscope V controller (Veeco, Santa Barbara, CA, USA). The analysis software uses the DMT model [40]. Torsional cantilevers (TL01, MikroMasch, Tallinn, Estonia) with a nominal spring constant of 2 N/m were used. The measured vertical and torsional resonance frequencies were 111 kHz and 1.1 MHz, respectively. The system was calibrated with a reference sample (model PS-LDPE, Veeco, Santa Barbara, CA, USA) [20]. Since HarmoniX assumes a spherical tip that indents an infinitely large and thick flat elastic surface, the value for the modulus of elasticity needs to be corrected offline. The first correction factor applied is to account for the different geometry, which in these experiments is a spherical tip indenting an infinitely long cylinder, see Figure 1. The correction factor used here is 2.1. The second correction factor was applied to account for the finite sample thickness of the protein fibril. A correction factor of 1.3, determined by the SPI measurements, was used.

### Peakforce QNM

Peakforce measurements were done on a Veeco Bioscope Catalyst microscope with a Nanoscope V controller (Veeco, Santa Barbara, CA, USA). The analysis software uses the DMT model [40]. The measurements were done both in ambient conditions (uncontrolled humidity, temperature, and air pressure) and physiological buffer (10 mM Tris-HCl, 50 mM NaCl, pH 7.4). The manufacturer provides a list of optimal cantilevers to measure specific ranges of elastic moduli. For the ambient measurements the stiff RTESP cantilevers (26.9 N/m, Veeco, Santa Barbara, CA, USA) were used, due to the high adhesion forces observed for other, less stiff cantilevers. For the measurements performed in buffer we used a medium stiff cantilever: FMR-10 cantilevers (nominal spring constant 2.8 N/m, Nanoworld, Neuchâtel, Switzerland). Here, the elastic moduli are also corrected offline as described for the HarmoniX data (see Harmonic force microscopy).

### Image analysis

Using SPIP software (Image Metrology A/S, Lyngby, Denmark), a trace was drawn on top of the fibril to determine the average height from the height images or modulus of elasticity from the stiffness maps of the individual fibrils, according to the procedure described in [4]. A point of potential confusion is that both HarmoniX and Peakforce QNM create so-called 'stiffness' maps, which in the software is expressed in units of Pa. Technically this is not correct, since stiffness is expressed in units of N/m. The parameter in these images is a modulus of elasticity which is expressed in Pa. In this manuscript we therefore refer to these values as moduli of elasticity. All images in this article are linewise corrected. Actual measurements are done on uncorrected images.

## Abbreviations

AFM: Atomic force microscopy; SPI: single-point nanoindentation.

## Competing interests

The authors declare that they have no competing interests.

## Authors' contributions

VS and MLB supervised the project, KKMS performed the research, and analyzed the results. KOW, MLB, and KKMS interpreted the results. All authors critically discussed the results and the manuscript.
